# Modeling Uterine Fibroids Using Bioengineered Hydrogels

**DOI:** 10.1021/acsbiomaterials.5c01026

**Published:** 2025-09-11

**Authors:** Allison K. Moses, Miriam Tamaño-Blanco, Erika Moore

**Affiliations:** Fischell Department of Bioengineering, 1068 University of Maryland, College Park, Maryland 20742, United States

**Keywords:** uterine fibroids, 3D in vitro model, fibroblast activation, extracellular matrix, transforming growth factor beta, hydrogel

## Abstract

Uterine fibroids are the most common gynecological tumors, characterized by excessive production of extracellular matrix. Despite their prevalence, the cellular mechanisms governing fibroid growth remain poorly understood. Current in vitro models for fibroids do not replicate the complex 3D tissue mechanics, structure, and extracellular matrix components of fibroids, which may limit our understanding of fibroid pathogenesis. To address this gap, we aimed to develop a 3D in vitro model to mimic aspects of the fibroid microenvironment. By encapsulating human uterine fibroblasts in poly­(ethylene glycol) (PEG)-based hydrogels comprising collagen- and fibronectin-derived peptides, this model allows for incorporation of fibroid cellular components, extracellular matrix components, and fibroid or myometrial tissue stiffness. Due to its mechanistic role in fibroblast activation and subsequent extracellular matrix production seen in fibroids, we treated uterine fibroblasts with transforming growth factor beta 3 (TGF-β3) to demonstrate quantification of fibrotic markers observed in fibroids. Here, we establish that human uterine fibroblasts increase α smooth muscle actin, extracellular matrix proteins, and cell elongation, as well as high metabolic activity and matrix remodeling in PEG-based hydrogels in response to TGF-β3. This research represents a physiologically relevant in vitro platform to investigate uterine fibroblast function within a 3D environment that mimics uterine fibroids, with the potential to advance our understanding of the cellular and molecular mechanisms driving fibroid growth and development.

## Graphical abstract

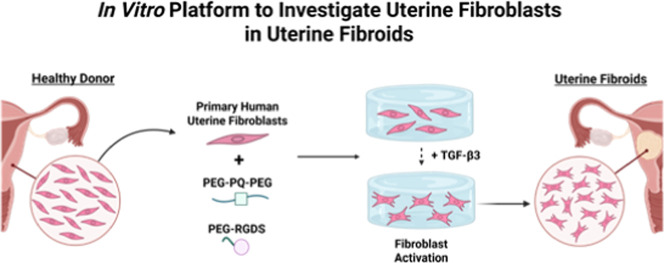

## 1 Introduction

Uterine fibroids, or uterine leiomyomas, are benign tumors that develop in the myometrial tissue of the uterus. Fibroids are the most common gynecological tumor in women,^1^ affecting up to 80% of African American women and 70% of European American women by age 50.^2^ Despite a high prevalence, there is no noninvasive cure for fibroids. Currently, the only curative treatment for fibroids is a hysterectomy, where the uterus is surgically removed.^3^ There exists a critical need for a safe, long-term, and nonsurgical treatment for uterine fibroids. However, current clinical options remain limited due to an incomplete understanding of fibroid pathogenesis and limited mechanistic insight into their formation.

Fibroids are composed of smooth muscle cells, fibroblasts, immune cells, and a predominantly collagen-based extracellular matrix (ECM). These cells and the ECM are regulated by growth factors, sex steroids, and chemokines and cytokines.^4–10^ Of the key cell types, fibroblasts are hypothesized to play a crucial role in fibroid development.^11^ Upon activation by key fibrogenic factors, such as transforming growth factor beta (TGF-β), fibroblasts can transform into myofibroblasts, which undergo uncontrolled and prolonged activation, where they proliferate and secrete excessive ECM,^12^ including collagen and fibronectin.^13^ The excessive production of ECM contributes to increased mechanical stiffness of the fibroid microenvironment compared to healthy myometrial tissue.^14–16^ Importantly, the TGF-β3 isoform is up to 5 times higher in fibroids compared to myometrial tissue.^11–13,17,18^ However, it is unclear whether elevated TGF-β3 acts as a driver of fibroid initiation or is a downstream consequence of other pathological changes in the tissue microenvironment. Despite these findings, significant gaps in knowledge remain regarding the mechanisms of fibroid growth and development.

Options to learn more about fibroids are currently limited to clinical samples and preclinical models. While clinical fibroid tissues are excellent resources to investigate tissue architecture and biological components of fibroids,^9,19^ they offer limited insight into mechanistic studies. Fresh tissue samples are difficult to attain and require access to multiple donor populations to enable statistical rigor, considering the disparities in fibroid prevalence, outcomes, and treatment approaches based on patient demographics.^20,21^ Unlike well-established tissue banks for research, such as the NIH Human Endometrial Tissue and DNA Bank,^22^ fibroid-specific tissue banks are still in development, with centers such as the Reproductive Subject Registry and Sample Repository led by Dr. Erica Marsh.^23^ Consequently, individual collections of fresh fibroid tissue samples are required for fibroid studies. Limited access to live fibroid samples, challenges with tissue viability after removal, and variability in tissue characteristics based on size, location, and patient demographics pose significant challenges to elucidate the cellular mechanisms that perpetuate disparities in prevalence.

Using isolated patient cells, uterine fibroid cell lines, or animal cells, researchers have characterized certain aspects of fibroid mechanisms. Current in vitro models for human fibroids research are 2D cell culture and 3D spheroid models. Commonly used biomaterials for fibroids research include Matrigel, collagen, and agarose.^24–26^ However, these current methods have limited ability to recapitulate key physiological aspects of the fibroid microenvironment, including essential ECM components, such as collagen and fibronectin, and the mechanical stiffness of fibroids while allowing for cell migration and nutrient diffusion. Our understanding of fibroblast activation in uterine fibroids remains limited due to the lack of physiologically relevant models for studying fibroid development outside of the body. Therefore, there is a critical need for a platform that can recapitulate key features of the fibroid microenvironment, including tissue stiffness and cell–matrix interactions, to enable mechanistic studies of disease progression.

Here, we established a physiologically relevant platform that mimics the fibroid microenvironment by leveraging a poly­(ethylene glycol) (PEG)-based hydrogel to characterize uterine fibroblast activation by TGF-β3. A physiologically relevant model allows us to mimic known variables seen in vivo, including bulk fibroid and myometrial mechanical properties, cell adhesion, and cell migration through the matrix. PEG-based hydrogel models have been widely used to mimic native ECM structure and mechanics.^27,28^ At controlled stiffnesses, PEG-based hydrogels can be modified for cell adhesion and matrix degradation sites using peptides derived from ECM proteins, specifically collagen and fibronectin. PEG-based hydrogels have also been used to assess fibroblast activation.^29–31^ We selected PEG-based hydrogels for this study due to their well-defined and tunable mechanical and biochemical properties. Unlike naturally derived matrices such as collagen or fibrin, PEG hydrogels allow independent control over stiffness and bioactivity through peptide incorporation. This modularity enables precise recapitulation of specific cues relevant to fibroid development while minimizing variability often associated with natural or composite hydrogels.^28^ Thus, a PEG-based hydrogel can be used to mimic native fibroid and myometrial tissue mechanical properties and ECM components to investigate fibroblast activation in fibroid development.

Leveraging a PEG-hydrogel system, we aimed to develop a physiologically relevant 3D in vitro model to study uterine fibroblast activation and mimic key aspects of the fibroid microenvironment. We hypothesized that exposure to TGF-β3 would activate uterine fibroblasts in our hydrogel system. In response to TGF-β3, human uterine fibroblasts demonstrated increased fibrotic activation, including hallmark fibrotic markers, in a fibroid-like environment. These results represent a 3D in vitro platform to investigate uterine fibroblast function within uterine fibroids, recapitulating the bulk mechanical stiffness and aspects of the ECM components in the uterine fibroid microenvironment. Together, our results demonstrate a 3D PEG-based hydrogel system to investigate uterine fibroid growth and development in a fibroid-like environment.

## 2 Materials and Methods

### 2.1 Cell Culture and Maintenance

Primary human uterine fibroblasts (HUFs) isolated from myometrial tissue of a 39 year-old woman were obtained from ATCC. HUFs were cultured in phenol-free, fibroblast basal medium supplemented with low serum growth kit (ATCC) and 1% 100× antibiotic-antimycotic (Gibco). HUF media was replaced every 48 h. Cells were maintained at 37 °C in 5% CO_2_ and passaged after 3–5 days before reaching 80% confluency. HUFs were used between passages 3–5 for hydrogel encapsulations.

### 2.2 PEG-Based Hydrogel Fabrication

Prior to hydrogel formation, acrylate-PEG-succinimidyl valerate (SVA) (acrylate-PEG-SVA) (MW: 3400 g/mol, Laysan Bio) was conjugated to a cell adhesive peptide RGDS (Arg-Gly-Asp-Ser) (MW: 433.42 g/mol, Fisher Scientific) and enzyme-cleavable peptide GGGPQGIWGQGK (PQ) (MW: 1141 g/mol, GeneScript) via amine substitution. The molar ratio of acrylate-PEG-SVA to RGDS was 1.2:1 to form PEG-RGDS, and the molar ratio of acrylate-PEG-SVA to PQ was 1:2 to form PEG–PQ–PEG. PEG peptides were conjugated in 20 mM *N*-(2-hydroxyethyl)­piperazine-*N*′-(4-butanesulfonic acid)) (HEPBS) buffer and 100 mM NaCl, 2 mM CaCl_2_, and 2 mM MgCl_2_ at a pH of 8.5. PEG peptides were titrated with 0.5 M NaOH to reach a pH of 8.0, mixed overnight at 4 °C, and dialyzed using 3.5 kDa molecular weight Spectra/Por dialysis membrane for PEG-RGDS and 6–8 kDa dialysis membrane for PEG–PQ–PEG. After 2 days of dialysis, PEG peptides were frozen at −80 °C, lyophilized, and returned to −80 °C until reconstitution for encapsulations.

### 2.3 Encapsulation of Cells

To encapsulate HUFs in PEG-based hydrogel, PEG-RGDS and PEG–PQ–PEG were reconstituted in HEPES buffer containing 10 mM HEPES and 100 mM NaCl with 1.5% triethanolamine (TEOA) (Sigma). The concentration of 2 mM PEG-RGDS was selected to optimize cell adhesion while enabling cell spreading. Then, concentrations of 4% or 6% (w/v) PEG–PQ–PEG were used to mimic myometrial and fibroid tissue stiffness, respectively, based on previous studies using PEG–PQ–PEG to modulate matrix stiffness.^32^ After reconstitution, 10 μM Eosin Y and 0.35% (v/v) *N*-vinylpyrrolidone (NVP) (Sigma) were mixed with the polymer solutions. HUFs were encapsulated in the PEG-based hydrogel at a cell density of 4 × 10^6^ cells/mL. Hydrogels were fabricated using a 5 μL droplet of the solution placed on a polydimethylsiloxane (PDMS) slab between two 380 μm PDMS spacers. A glass coverslip modified with methacrylate groups to attach to the hydrogel was placed on top of the hydrogel droplet. The coverslip, droplet, and PDMS were exposed to white light for 1 min to enable the cross-linking reaction. Hydrogels with the attached coverslips were placed into a 24-well plate face up, and 1 mL of media was given to each well.

### 2.4 Compressive Testing of Hydrogels

Hydrogel Young’s Modulus was determined by compressive testing using an Instron 5942 (Universal Testing Systems). Acellular 4% or 6% PEG–PQ–PEG and 2 mM PEG-RGDS hydrogels were fabricated as described previously, using 74 μL droplets in a PDMS mold to achieve approximately 3.0 mm in height and 5.6 mm in diameter. To account for the increased hydrogel height relative to the previously used 5 μL droplets, hydrogels were cross-linked under white light for 7 min and 53 s to ensure a comparable degree of cross-linking. Acellular hydrogels were soaked in PBS for 24 h to allow swelling prior to compression testing. Compression testing was performed at a uniaxial ramp rate of 0.003 mm/s at room temperature. The resultant stress–strain data was plotted, and Young’s modulus was recorded from the slope of the linear region of the stress–strain plots from three hydrogels per group. Similarly, cells were encapsulated at a density of 4 × 10^6^ cells/mL in the PEG–PQ–PEG and PEG-RGDS hydrogels, and compression testing was performed at 24 h and 7 days to determine the Young’s modulus over time.

### 2.5 TGF-β3 Treatments on Encapsulated Cells

TGF-β3-treated media was made using lyophilized TGF-β3 (Thermo Fisher) dissolved in HUF media to reach a stock concentration of 200 ng/mL TGF-β3. Immediately after encapsulation, HUFs were treated with TGF-β3 treated HUF media at a concentration of either 10 ng/mL, 20 ng/mL, or 0 ng/mL (no treatment control). Encapsulated HUFs were cultured for up to 7 days with corresponding media changes at every 48 h.

### 2.6 TGF-β Inhibition of Encapsulated Cells

TGF-β inhibitor media was made by diluting 10 mM SB-431542 in 1 mL DMSO (MedChemExpress) to 10 μM SB-431542 (0.1% DMSO) in HUF media. Immediately after encapsulation, HUFs were treated with the following: no treatment, 20 ng/mL TGF-β3, 20 ng/mL TGF-β3 and 10 μM SB-431542, 10 μM SB-431542, and 0.1% DMSO (vehicle control) in HUF medias. Encapsulated HUFs were cultured for 4 days, with corresponding media changes after 48 h.

### 2.7 Immunocytochemistry

Immunocytochemistry was performed to assess HUF proliferation in the hydrogels and HUF response to TGF-β3 treatments. HUFs were fixed with 4% paraformaldehyde for 40 min at room temperature, rinsed 3 times with PBS for 5 min intervals, and stored in 0.2% sodium azide until further use. Cells were then permeabilized with 0.25% Triton-X for 45 min, rinsed 4 times with PBS for 10 min intervals, blocked in 5% donkey serum in PBS at 4 °C overnight, and rinsed 3 times with PBS for 5 min intervals. Primary antibodies were added to the hydrogels in 0.5% donkey serum in PBS at a dilution of 1:200 for 2 nights. Hydrogels were rinsed with 0.01% Tween in PBS 4 times for 1 h intervals. Hydrogels were rinsed with PBS for 1 h before secondary antibodies at a dilution of 1:400 in PBS were added for 2 nights at 4 °C. Hydrogels were rinsed in PBS once for 3 h. Overnight, 2 μM 4′,6-diamidino-2-phenylindole (DAPI) was used to stain cell nuclei. Hydrogels were rinsed 3 times in PBS before imaging.

For assessing cell activation response to TGF-β3 treatments, hydrogels were fixed at 7 days. The following primary and secondary antibodies were used: α smooth muscle actin (Mouse-Anti-Human Monoclonal Antibody, Abcam), collagen I (Rabbit-Anti-Human Recombinant Monoclonal Antibody, Abcam), AlexaFluor 488 (Donkey Anti-Rabbit, Thermo Fisher), and AlexaFluor 555 (Donkey Anti-Mouse, Thermo Fisher). Additionally, AlexaFluor Phalloidin 647 (Thermo Fisher) (1:60 dilution in DAPI) was used to stain F-actin to visualize cell bodies.

Because media changes occurred every 48 h, a time course at the conclusion of each media change was conducted. To assess collagen production and proliferation over time with or without TGF-β3 treatment, hydrogels were fixed at 24 h, 4 days, and 6 days. The following primary and secondary antibodies were used: collagen I (Rabbit-Anti-Human Recombinant Monoclonal Antibody, Abcam), collagen III (Mouse-Anti-Human Monoclonal Antibody, Abcam), Ki67 (Chicken-Anti-Human Polyclonal Antibody, Abcam), AlexaFluor 488 (Donkey Anti-Rabbit, Thermo Fisher), AlexaFluor 555 (Donkey Anti-Mouse, Thermo Fisher), AlexaFluor 647 (Donkey Anti-Chicken, Thermo Fisher).

To assess cell activation response to TGF-β3 and TGF-β inhibition, hydrogels were fixed at 4 days. The following primary and secondary antibodies were used: α smooth muscle actin (Mouse-Anti-Human Monoclonal Antibody, Abcam), collagen I (Rabbit-Anti-Human Recombinant Monoclonal Antibody, Abcam), AlexaFluor 488 (Donkey Anti-Rabbit, Thermo Fisher), and AlexaFluor 555 (Donkey Anti-Mouse, Thermo Fisher). Additionally, AlexaFluor Phalloidin 647 (Thermo Fisher) (1:60 dilution in DAPI) was used to stain F-actin to visualize cell bodies.

### 2.8 Enzyme-Linked Immunosorbent Assay (ELISA) for TGF-β3

A sandwich SimpleStep ELISA (Abcam) was performed according to manufacturer recommendations to detect TGF-β3. Conditioned media was collected from nontreated and TGF-β3-treated hydrogels at 6 days and frozen at −80 °C until further use. The samples were thawed once and centrifuged at 2000*g* for 10 min. For each experimental group, conditioned media from three independent hydrogels and analyzed individually. The TGF-β3 standards were diluted from 16 ng/mL to 0 ng/mL in duplicates to generate a standard curve. In duplicates, fresh nontreated media and fresh TGF-β3-treated media were used as controls. The capture antibody was added to each well and incubated for 1 h at room temperature on a plate shaker set to 400 rpm. Samples were washed three times with 1× wash buffer PT, and all excess liquid was removed. To visualize the presence of TGF-β3 in each well, tetramethylbenzidine (TMB) solution was added to each well for 10 min in the dark on the plate shaker (400 rpm), and stop solution was added to each well for 1 min. A Spark Microplate Reader (Tecan) was used to measure the optical density at an absorbance of 450 nm. Total target protein levels in each sample were determined by interpolation from the standard curve.

### 2.9 Glycolysis Assay

A glycolysis assay was performed using the Glycolysis Assay Kit (Millipore Sigma) according to manufacturer recommendations to measure the production of l-lactate. Conditioned media was collected from nontreated and TGF-β3-treated hydrogels at 6 days and frozen at −80 °C until further use. Media samples were thawed once and centrifuged at 2000*g* for 10 min. For each experimental group, conditioned media from three independent hydrogels and analyzed individually. The l-lactate standards were diluted from 10 mM to 0 mM in fresh nontreated media in duplicates to generate a standard curve. In duplicates, fresh TGF-β3-treated media was also used as a control. A working reagent consisting of assay buffer, enzymes A and B, and NAD/MTT was added to each well. The samples were incubated at room temperature for 30 min. Using a Spark Microplate Reader (Tecan), absorbance was measured at 565 nm, and sample concentrations were calculated by interpolation from the standard curve. l-Lactate concentrations in the TGF-β3-treated groups were normalized to the l-lactate concentration in the fresh TGF-β3-treated media.

### 2.10 Western Blot of Lysyl Oxidase

Western blot was performed to quantify the expression of lysyl oxidase (LOX) protein. Proteins from hydrogel-encapsulated cells were obtained by snap freeze of hydrogels in liquid nitrogen. Four hydrogels were used per treatment group. Cell lysis was achieved with RIPA buffer, including Halt protease inhibitor cocktail (Thermo Fisher Scientific) followed by centrifugation at 13,000*g* for 15 min. Protein concentrates were quantified by Pierce BCA assay (Thermo Fisher Scientific), 10 μg of the protein samples were denatured with NuPAGE LDS sample buffer containing NuPAGE sample reducing agent and boiled at 70 °C for 10 min prior to use. Proteins were separated by gel electrophoresis using NuPAGE Novex 4–12% Bis-Tris precast gels and transferred onto PVDF membranes using the Trans-Blot Turbo Transfer System (Bio-Rad). Membranes were blocked with 4% nonfat milk in 0.2% Tween 20 PBS for 30 min at room temperature and probed overnight at 4 °C with the primary antibody, Lysyl oxidase LOX (1:1000 mouse monoclonal MA5-25033 Thermo Fisher Scientific). Next, membranes were washed and incubated with secondary antibody (1:1000 Mouse IgG Horseradish Peroxidase-conjugated Antibody, RD Systems), for 1 h at room temperature. Western detection was performed using SuperSignal West Pico PLUS Substrate (Thermo Fisher Scientific). Immunoreactive bands were visualized in FluorChem E Gel Imaging System (Bio-Techne).

### 2.11 Image Analysis

HUF cell characteristics, including protein expression and morphology, were determined using IMARIS software to analyze immunocytochemistry images. For all IMARIS analysis, 3 images from each of the 3 hydrogels in each treatment group were used for analysis. Using IMARIS spot analysis, the number of DAPI positive cells were totaled for each image.

To assess cell morphology and clustering, cell bodies were stained with Phalloidin (F-actin). Due to frequent overlap of cell bodies in the images, the total number of DAPI-stained nuclei was compared to the total number of segmented cell bodies, referred to as cell segments. The ratio of cell nuclei to cell segments was used to infer cell organization. A ratio near 1 suggests that most segments contain a single cell nucleus, which indicates more separated or rounded cells. A ratio greater than 1 suggests increased cell overlap or clustering, where multiple nuclei reside within a single segment, indicating more elongated or interconnected structures.

Using IMARIS surface analysis, the total volume of αSMA, Col1, or Col3 proteins were normalized to the number of DAPI positive cells in each image to determine the average cell expression of αSMA, Col1, or Col3.

To determine the percentage of proliferating cells in each image, IMARIS surface analysis was applied to count the number of cells expressing the nuclear marker, Ki67, compared to the total number of cells.

For LOX expression, densitometric analysis on Western Blot membranes was performed using Fiji (ImageJ) software to determine the area under the curve for each band. The area under the curves was plotted as a band intensity for LOX expression.

### 2.12 Statistical Analysis

Statistical analysis and graphing were performed using GraphPad Prism software. Acellular compression testing, ELISA, and glycolysis assays were analyzed using an unpaired *t*-test, and compression testing experiments with encapsulated cells were analyzed using two-way ANOVA with Tukey’s post hoc tests. Immunocytochemistry experiments for αSMA, Col1, and cell nuclei to segment ratios were analyzed using one-way ANOVA followed by Tukey’s post hoc tests after confirming normality. Immunocytochemistry experiments for Col1, Col3, and Ki67 over time were analyzed using two-way ANOVA followed by Tukey’s post hoc tests. Sample sizes are reported in each figure legend. All data is shown as mean ± standard deviation.

## 3 Results

### 3.1 Bulk Mechanical Properties of Myometrial- and Fibroid-Mimicking Hydrogels

Figure 1A depicts the HUFs encapsulation in the PEG-based hydrogel. Acellular hydrogels were subjected to compression testing to quantify bulk mechanical properties. Modulating the concentration of PEG–PQ–PEG with 2 mM PEG-RGDS, acellular hydrogels composed of 4% PEG–PQ–PEG had an average Young’s modulus of 2.2 ± 0.2 kPa, while 6% PEG–PQ–PEG hydrogels were significantly stiffer, with an average 6.1 ± 0.5 kPa (Figure 1B). To determine how hydrogel stiffness changes with the incorporation of cells, HUFs were encapsulated in the 4% PEG–PQ–PEG and 6% PEG–PQ–PEG hydrogels. At 24 h and 7 days, 4% PEG–PQ–PEG hydrogel stiffness was 1.6 kPa and 1.3 ± 0.1 kPa, whereas 6% PEG–PQ–PEG stiffness decreased from 4.6 ± 0.8 kPa to 3.8 ± 0.3 kPa (Figure 1C).

**Figure 1:**
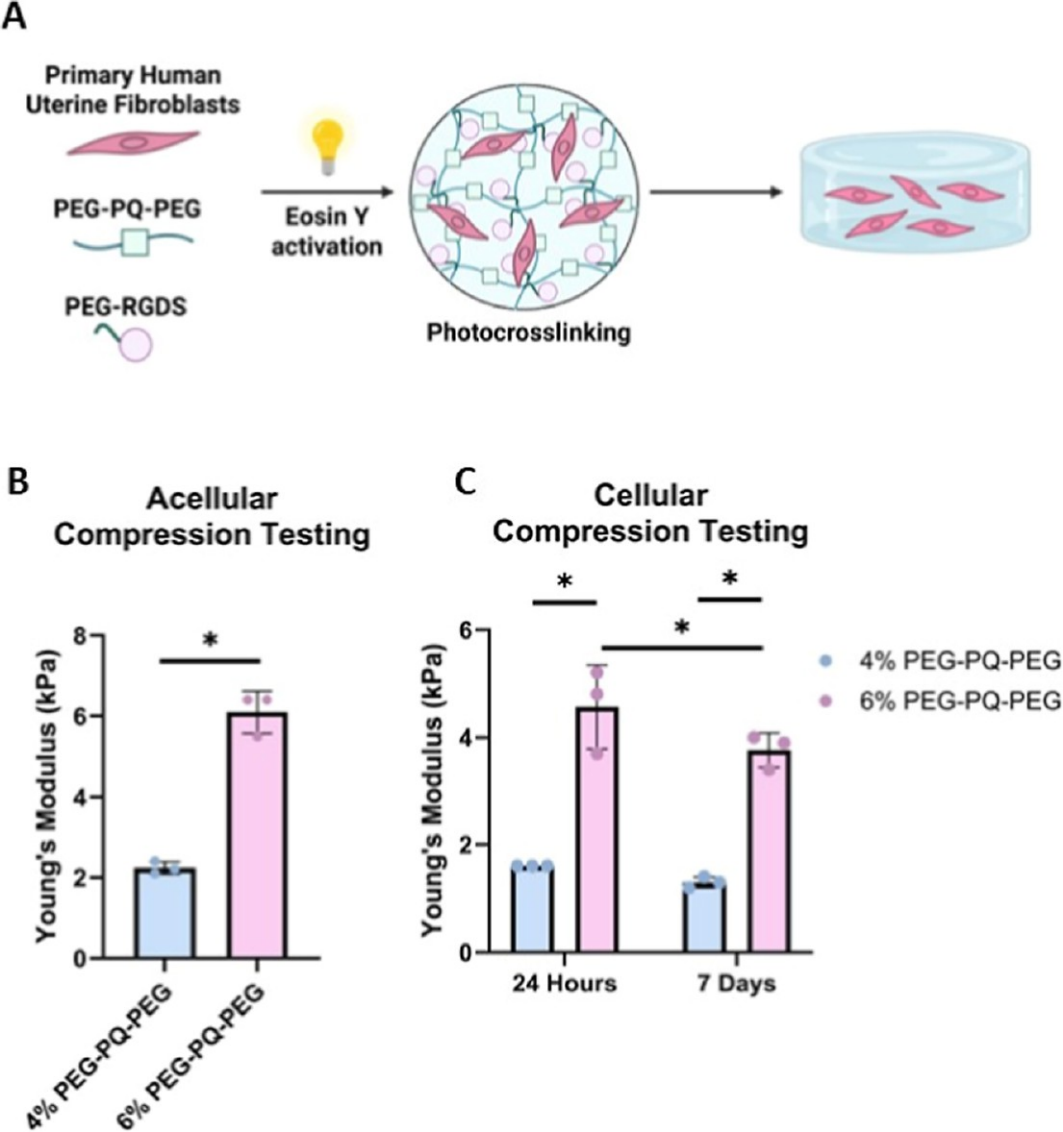
PEG-based hydrogels mimic bulk mechanical properties of myometrial and fibroid tissue with and without encapsulated human uterine fibroblasts. (A) Schematic illustration of the poly­(ethylene glycol) (PEG)-based hydrogel, where primary human uterine fibroblasts (HUFs) are encapsulated into a PEG–PQ–PEG and PEG-RDGS hydrogel by photo-cross-linking in the presence of Eosin Y/NVP activation. (B) With compression testing, the Young’s modulus of acellular 4% PEG–PQ–PEG and 6% PEG–PQ–PEG hydrogels were determined to be 2.2 ± 0.2 kPa and 6.1 ± 0.5 kPa, respectively. *N* = 3 hydrogels per group. (C) Compression testing of HUFs encapsulated in 4% PEG–PQ–PEG and 6% PEG–PQ–PEG hydrogels after 24 h and 7 days. *N* = 3 hydrogels per group at each time point; * = *p* < 0.05. Data expressed as mean ± standard deviation.

### 3.2 α-Smooth Muscle Actin, Collagen I, and Cell Elongation with TGF-β3 in Myometrial- and Fibroid-Mimicking Hydrogels

We characterized changes in fibroblast activation, ECM production, and morphology after treatment with TGF-β3 in both hydrogel stiffnesses after 7 days of encapsulation (Figures 2 and 3). To visualize the HUFs that were activated within the hydrogel matrices, we stained for α-smooth muscle actin (αSMA). We also quantified collagen I (Col1) expression to assess ECM production. Additionally, HUF cell morphology was assessed via F-actin (Phalloidin) staining (Figures 2A, 3A, Supporting Information Figure 1A).

**Figure 2:**
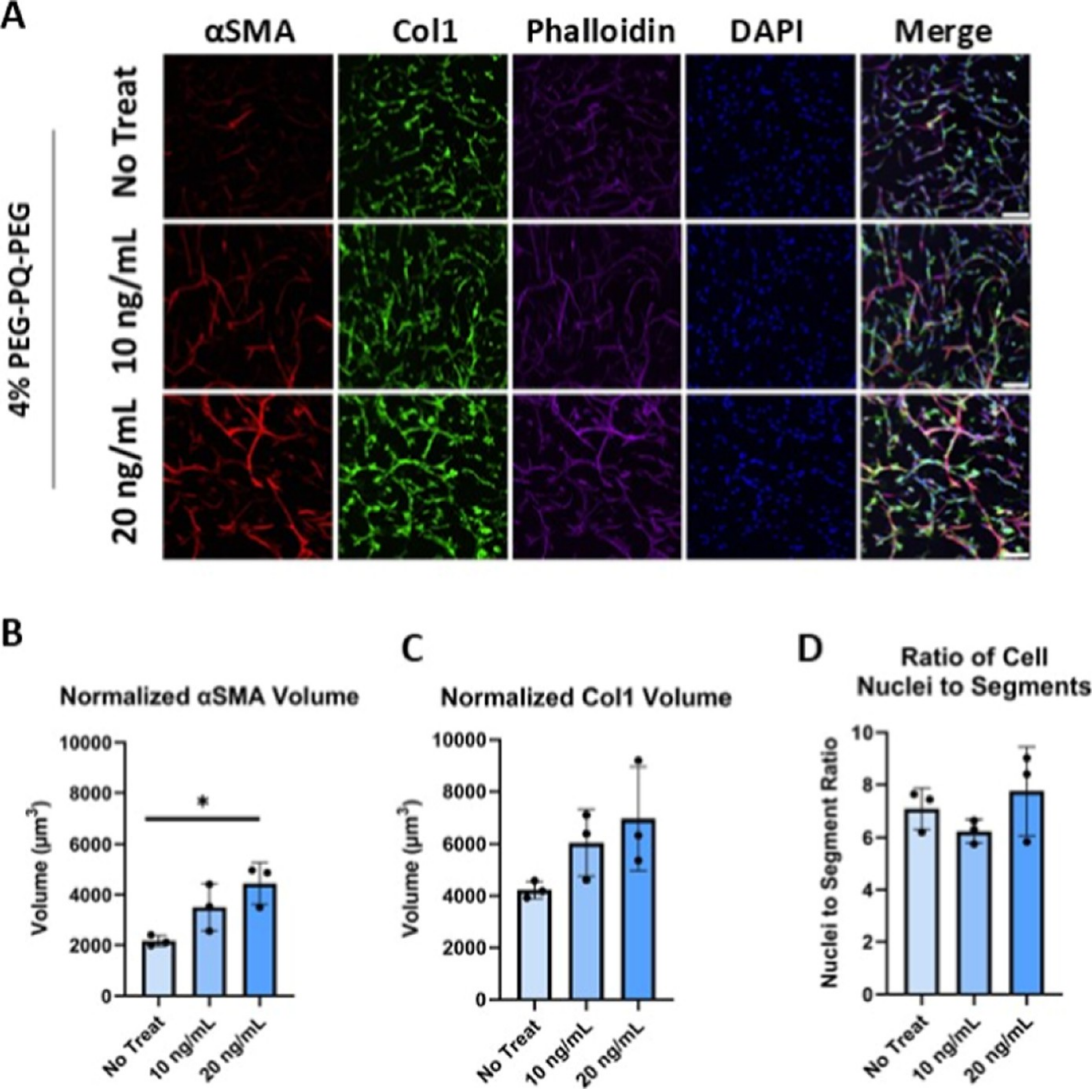
Human uterine fibroblasts increase αSMA expression in response to TGF-β3 treatment in the 4% PEG–PQ–PEG myometrial-mimicking hydrogels. (A) Representative images of HUF expression of α smooth muscle actin (αSMA in red), collagen I (Col1 in green), Phalloidin via F-actin (purple), nuclei (DAPI in blue), and merged in 4% PEG–PQ–PEG hydrogels treated with 10, 20 ng/mL TGF-β3 or no treatment controls. (B) Total αSMA volume normalized to DAPI counts, where 20 ng/mL TGF-β3 increases average αSMA expression. (C) Total Col1 volume normalized to DAPI counts. (D) Ratio of cell nuclei to cell segments to determine cell clustering or elongation. All scale bars = 100 μm; 20× magnification; *N* = 3 technical replicates; * = *p* < 0.05. Data expressed as mean ± standard deviation.

**Figure 3:**
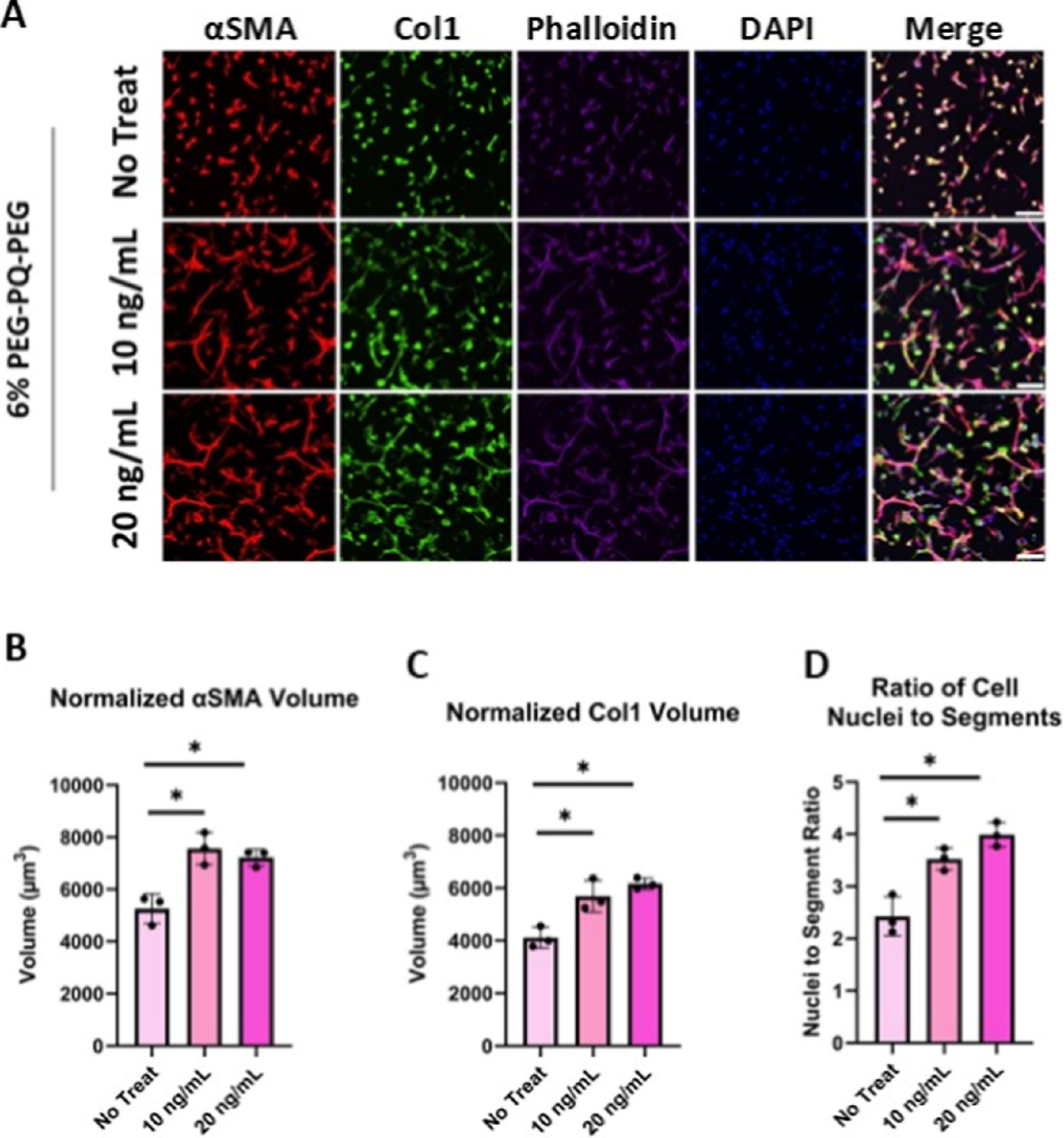
Human uterine fibroblasts increased αSMA, collagen I and cell elongation in response to TGF-β3 treatments in the 6% PEG–PQ–PEG fibroid-mimicking hydrogels. (A) Representative images of HUF expression of α smooth muscle actin (αSMA in red), collagen I (Col1 in green), Phalloidin via F-actin (purple), nuclei (DAPI in blue), and merged in 6% PEG–PQ–PEG hydrogels treated with 10, 20 ng/mL TGF-β3 or no treatment controls. (B) Total αSMA volume normalized to DAPI counts, where 10 and 20 ng/mL TGF-β3 increases average αSMA expression. (C) Total Col1 volume normalized to DAPI counts, where 10 and 20 ng/mL TGF-β3 increases average Col1 expression. (D) Ratio of cell nuclei to cell segments to determine cell clustering or elongation, where 10 and 20 ng/mL TGF-β3 increases elongation. All scale bars = 100 μm; 20× magnification; *N* = 3 technical replicates; * = *p* < 0.05. Data expressed as mean ± standard deviation.

Upon treatment with 20 ng/mL TGF-β3, HUF expression of αSMA increased in 4% PEG–PQ–PEG hydrogels compared to nontreated hydrogels. However, treatment with 10 ng/mL TGF-β3 did not significantly affect αSMA expression compared to both nontreated and 20 ng/mL hydrogels (Figure 2B). Although nonsignificant, increased TGF-β3 treatments tended to increase Col1 in 4% PEG–PQ–PEG hydrogels (Figure 2C). Cell morphology was assessed by calculating the ratio of cell nuclei to segmented cell structures, where a higher ratio indicates greater cell elongation. TGF-β3 treatment did not significantly alter this ratio in the 4% PEG–PQ–PEG hydrogels, although all groups exhibited a high degree of elongation (Figure 2D).

In the 6% PEG–PQ–PEG hydrogels, 10 ng/mL and 20 ng/mL TGF-β3 treatments significantly increased αSMA, Col1, and cell elongation compared to no treatment. There were no significant differences in αSMA, Col1, nor cell elongation between 10 ng/mL and 20 ng/mL TGF-β3 treatments in the 6% PEG–PQ–PEG hydrogels (Figure 3B–D). Taken with the observed increase in αSMA expression in 4% PEG–PQ–PEG hydrogels following treatment with 20 ng/mL TGF-β3, we selected 20 ng/mL as the treatment concentration of TGF-β3 for subsequent experiments.

### 3.3 Cell Proliferation with TGF-β3 Treatment over Time

We then assessed HUF cell proliferation in the PEG-based hydrogels using the Ki67 nuclear marker over time (Figure 4, Supporting Information Figure 1B). In the 4% PEG–PQ–PEG hydrogels (Figure 4A), 20 ng/mL TGF-β3 treatment had no effect on cell proliferation at any time point compared to nontreated groups. However, in both nontreated and TGF-β3-treated groups, the average percent cell proliferation decreased from 68.53% and 57.29% to 12.86% and 9.75% from 24 h to 4 days, respectively. From day 4 to day 6, cell proliferation in the nontreated hydrogel increased to 40.18% but remained significantly lower than at 24 h. By 6 days, TGF-β3-treated cell proliferation remained low at 30.78% (Figure 4B).

**Figure 4:**
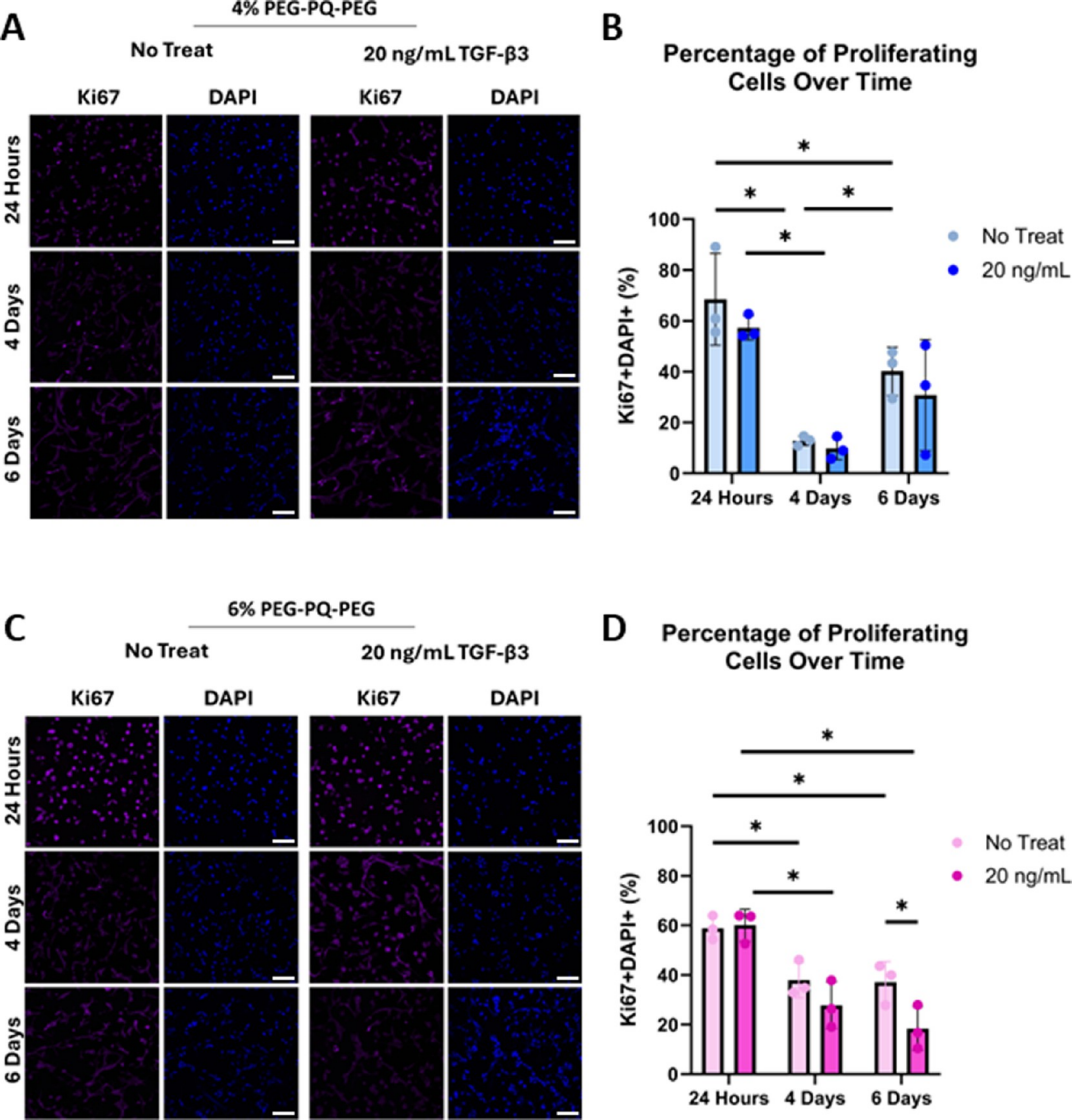
Uterine fibroblasts decrease proliferation in response to TGF-β3 over time in the hydrogels mimicking myometrial and fibroid stiffness. (A) Representative images of HUF expression of proliferation marker, Ki67 (in purple), and cell nuclei (DAPI in blue) in 4% PEG–PQ–PEG hydrogels treated with 20 ng/mL TGF-β3 or no treatment controls. (B) Percent of Ki67 positive cells at 24 h, 4 days, and 6 days with or without TGF-β3 treatment in 4% PEG–PQ–PEG. (C) Representative images of HUF expression of Ki67 (purple) and DAPI (blue) in 6% PEG–PQ–PEG hydrogels treated with 20 ng/mL TGF-β3 or no treatment controls. (D) Percent of Ki67 positive cells at 24 h, 4 days, and 6 days with or without TGF-β3 treatment in 6% PEG–PQ–PEG. All scale bars = 100 μm; 20× magnification; *N* = 3 technical replicates at each time point; * = *p* < 0.05. Data expressed as mean ± standard deviation.

In the 6% PEG–PQ–PEG hydrogels (Figure 4C), cell proliferation significantly decreased in both nontreated and TGF-β3-treated groups from 58.91% and 60.09% to 37.98% and 27.81% from 24 h to 4 days, respectively. However, TGF-β3 had no significant effect on cell proliferation from 24 h to 4 days. By day 6, cell proliferation in the TGF-β3-treated hydrogels remained low at 18.41% but was significantly reduced compared to the 37.14% cell proliferation with no treatment (Figure 4D).

### 3.4 Collagen I & III Expression with TGF-β3 Treatment over Time

We performed immunocytochemical staining for Col1 and Col3 (Figure 5, Supporting Information Figure 1B). In the 4% PEG–PQ–PEG hydrogels (Figure 5A), we observed no significant differences in Col1 nor Col3 over time with or without TGF-β3 treatment, although Col1 tended to increase with TGF-β3 treatment compared to no treatment at each time point (Figure 5B,C). Unlike Col1 expression in the 4% PEG–PQ–PEG hydrogels, in the 6% PEG–PQ–PEG hydrogels (Figure 5D), Col1 volume significantly increased with TGF-β3 treatment at each time point. Additionally, with TGF-β3 treatment, Col1 significantly increased from 24 h to 4 days and decreased from 4 days to 6 days (Figure 5E). Like Col3 expression in the 4% PEG–PQ–PEG hydrogels, there were no significant differences in Col3 over time with or without TGF-β3 treatment (Figure 5F). Lastly, in both hydrogels, there appeared to be greater expression of Col1 compared to Col3.

**Figure 5:**
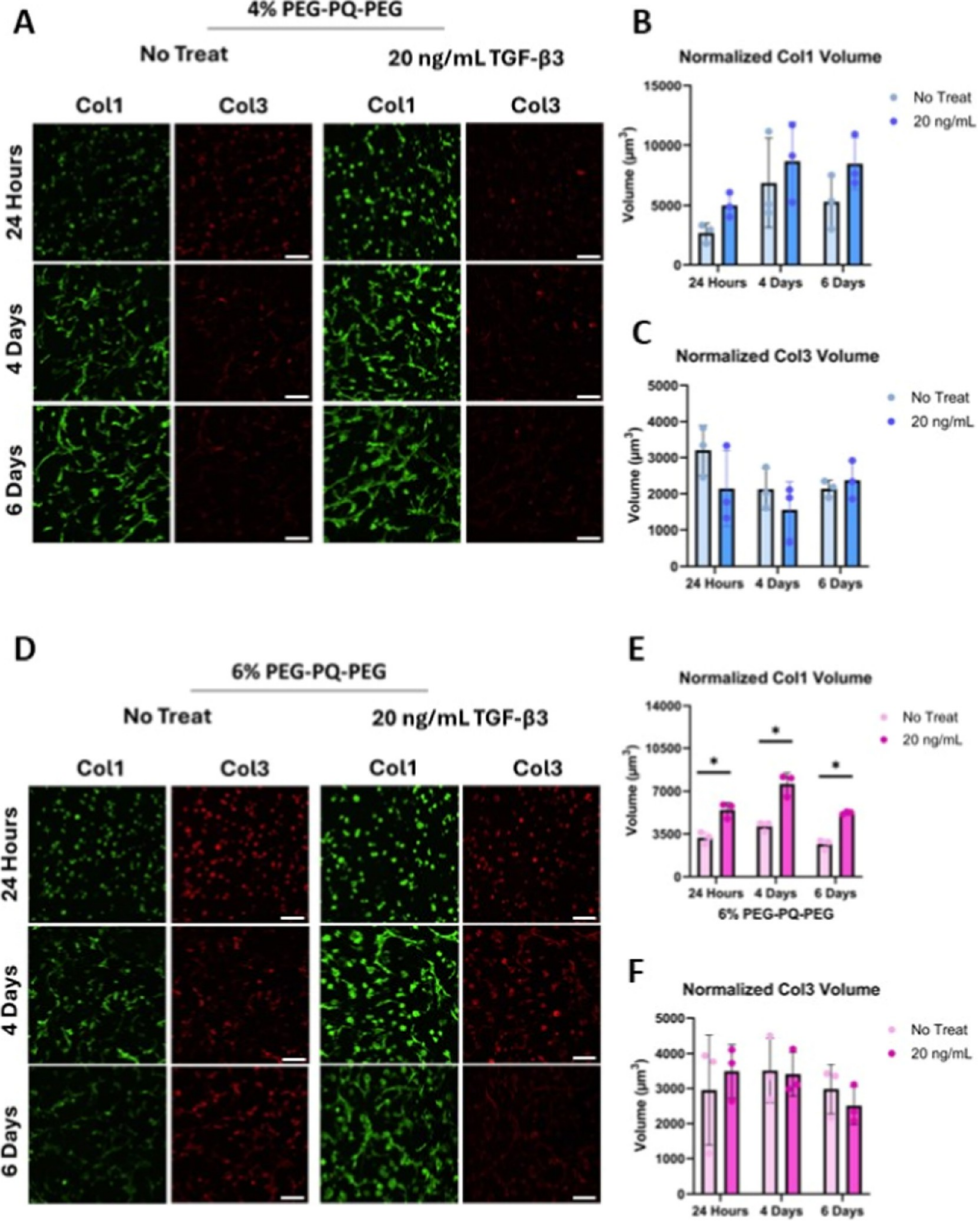
TGF-β3 increases collagen I expression in 6% PEG–PQ–PEG fibroid-mimicking hydrogels but not in 4% PEG–PQ–PEG myometrial-mimicking hydrogels while no significant changes are seen in collagen III expression. (A) Representative images of HUF expression of collagen I (Col1 in green) and collagen III (Col3 in red) in 4% PEG–PQ–PEG hydrogels treated with 20 ng/mL TGF-β3 or no treatment controls. (B) Total Col1 volume normalized to DAPI counts at 24 h, 4 days, and 6 days with or without TGF-β3 treatment in 4% PEG–PQ–PEG. (C) Total Col3 volume normalized to DAPI counts at 24 h, 4 days, and 6 days with or without TGF-β3 treatment in 4% PEG–PQ–PEG. (D) Representative images of HUF expression of Col1 in green and Col3 in red in 6% PEG–PQ–PEG hydrogels treated with 20 ng/mL TGF-β3 or no treatment controls. (E) Total Col1 volume normalized to DAPI counts at 24 h, 4 days, and 6 days with or without TGF-β3 treatment in 6% PEG–PQ–PEG. (F) Total Col3 volume normalized to DAPI counts at 24 h, 4 days, and 6 days with or without TGF-β3 treatment in 6% PEG–PQ–PEG. All scale bars = 100 μm; 20× magnification; *N* = 3 technical replicates at each time point; * = *p* < 0.05. Data expressed as mean ± standard deviation.

### 3.5 Glycolysis and Lysyl Oxidase Expression in Response to TGF-β3 Treatment

We characterized differences in HUF cell metabolism with or without TGF-β3 treatment in both matrix stiffnesses at 6 days (Figure 6). First, to assess TGF-β3 uptake by HUFs, we quantified its concentration in conditioned media collected at day 6, or 48 h after the second media exchange. The 6% PEG–PQ–PEG hydrogels exhibited significantly lower levels of TGF-β3 in the conditioned media (0.35 ng/mL) compared to 4% PEG–PQ–PEG (0.55 ng/mL). Fresh media was assessed as a control, measuring a TGF-β3 concentration of 0.04 ng/mL, significantly less than TGF-β3-treated conditioned media groups (Figure 6A). The presence of TGF-β3 in nontreated conditioned media hydrogel groups was below the detectable limit.

**Figure 6:**
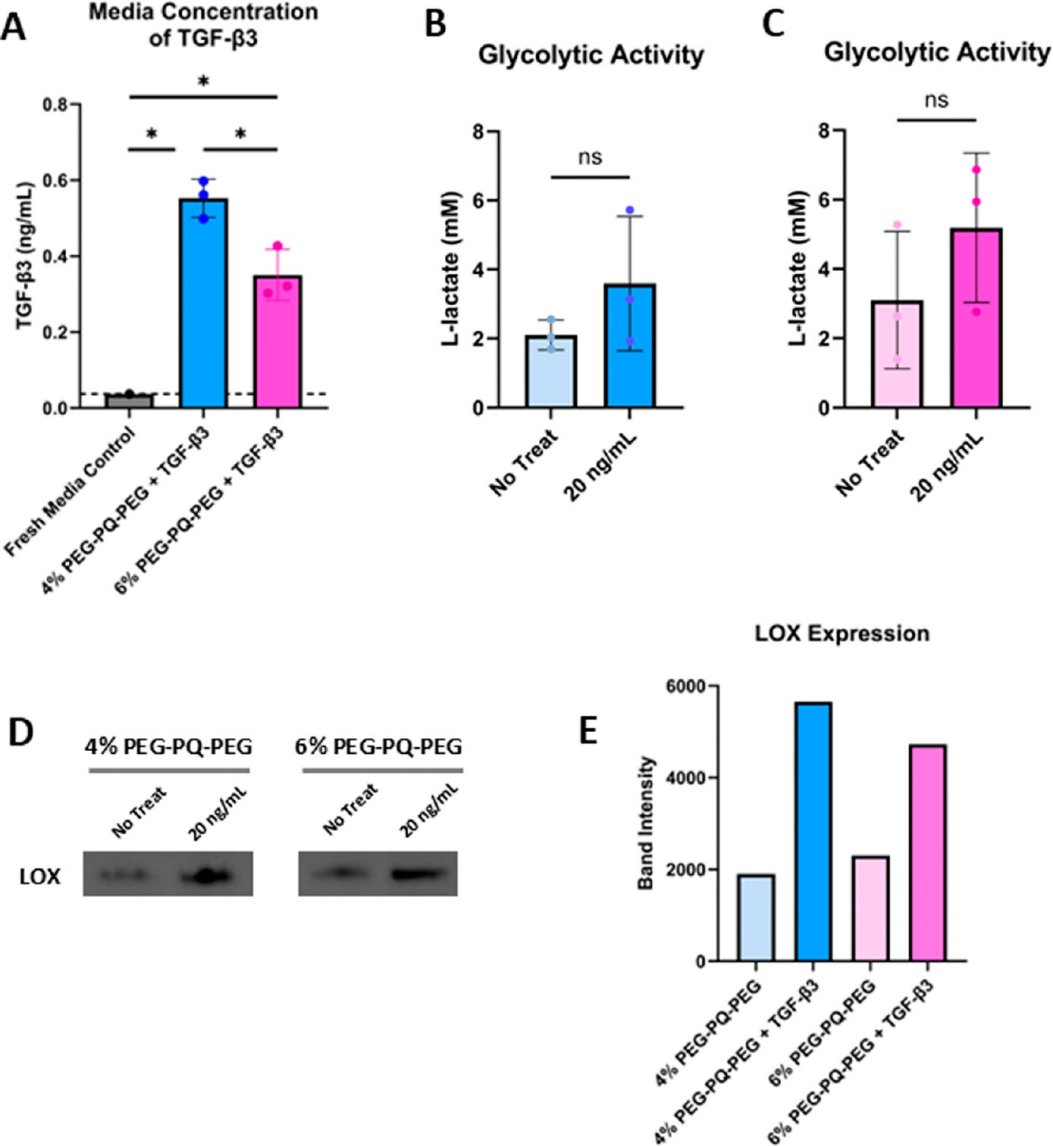
TGF-β3 treatment increases LOX expression in both hydrogels. (A) TGF-β3 concentration in cell-conditioned media of 20 ng/mL TGF-β3-treated hydrogels at day 6, with fresh media as a control. (B) Concentration of l-lactate in cell-conditioned media from 4% PEG–PQ–PEG, myometrial-mimicking hydrogels treated with or without 20 ng/mL TGF-β3 at day 6. (C) Concentration of l-lactate in cell-conditioned media from 6% PEG–PQ–PEG, fibroid-mimicking hydrogels treated with or without 20 ng/mL TGF-β3 at day 6. (D) Western blot for LOX in both 4% and 6% PEG–PQ–PEG hydrogels with or without TGF-β3 treatment at day 7, with 4 hydrogels per group. (E) Quantification of LOX band intensity from Western blot of both hydrogels with and without TGF-β3 treatment. *N* = 3 technical replicates for cell-conditioned media experiments; * = *p* < 0.05. Data expressed as mean ± standard deviation.

We then determined HUF cell glycolytic activity by quantifying the concentration of l-lactate present in conditioned media normalized to fresh and TGF-β3-treated media. With or without TGF-β3, HUFs produced high levels of l-lactate at 6 days in both myometrial-mimicking and fibroid-mimicking hydrogels. While not statistically significant, we observed slight increases in the average l-lactate in the TGF-β3-treated hydrogel groups (Figure 6B,C).

Next, we quantified lysyl oxidase (LOX) protein expression with Western blotting to further characterize HUF cell activation after 7 days of encapsulation with or without TGF-β3 (Figure 6D). We observed increased LOX protein expression in both the myometrial-mimicking hydrogels and fibroid-mimicking hydrogels when treated with 20 ng/mL TGF-β3. In the myometrial-mimicking hydrogels, TGF-β3 treatment elicited a 2.97-fold increase in LOX expression. Similarly, TGF-β3 treatment elicited a 2.05-fold increase in LOX expression in the fibroid-mimicking hydrogels (Figure 6E).

### 3.6 TGF-β Signaling Inhibition

Lastly, we inhibited TGF-β signaling to confirm that the observed effects are mediated by TGF-β3 (Figure 7). To do so, we performed immunocytochemical staining for αSMA, Col1, and F-actin (via Phalloidin) in response to treatment with the TGF-β receptor-1 kinase inhibitor, SB-431542 (Figure 7A, Supporting Information Figure 1C). Due to the observed changes in Col1 expression by 4 days (Figure 5E), we quantified Col1 expression after 4 days of encapsulation in 6% PEG–PQ–PEG hydrogels. We observed that 10 μM SB-431542 alone and in combination with 20 ng/mL TGF-β3 treatment significantly reduced Col1 expression compared to no treatment and 20 ng/mL TGF-β3 treatment. There was no statistically significant difference between no treatment and the vehicle control (0.1% DMSO). Additionally, there was no significant difference between 10 μM SB-431542 alone and 10 μM SB-431542 in combination with 20 ng/mL TGF-β3 treatment (Figure 7B).

**Figure 7:**
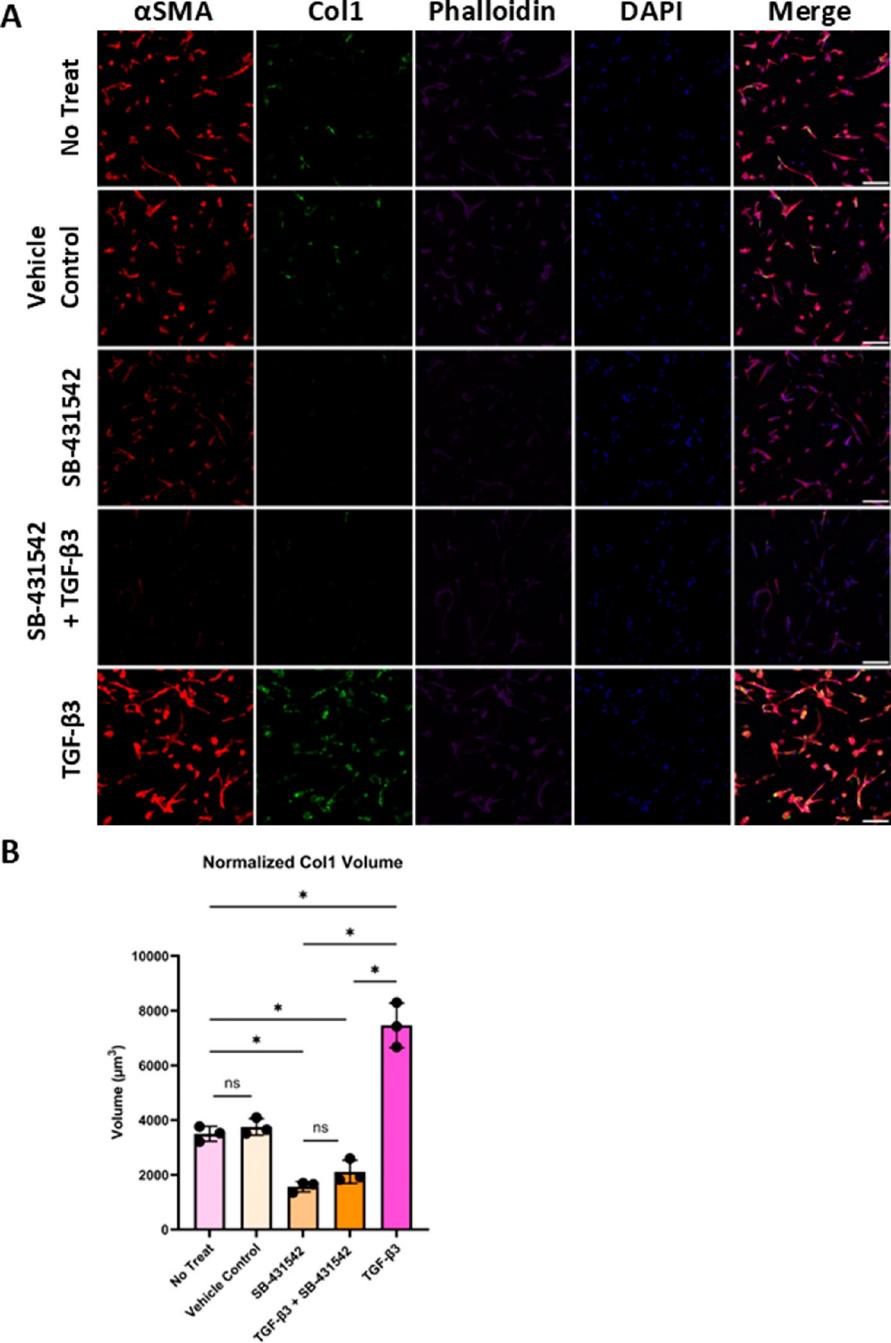
Inhibition of TGF-β signaling confirms TGF-β3-specific response in the 6% PEG–PQ–PEG fibroid-mimicking hydrogels at 4 days. (A) Representative images of HUF expression of α smooth muscle actin (αSMA in red), collagen I (Col1 in green), Phalloidin via F-actin (purple), and nuclei (DAPI in blue) in 6% PEG–PQ–PEG hydrogels treated with no treatment, 0.1% DMSO (vehicle control), 10 μM SB-431542, 20 ng/mL TGF-β3 and 10 μM SB-431542, or 20 ng/mL TGF-β3. (B) Total Col1 volume normalized to DAPI counts. All scale bars = 100 μm; 20× magnification; *N* = 3 technical replicates; * = *p* < 0.05. Data expressed as mean ± standard deviation.

## 4 Discussion

Uterine fibroids are the most common benign gynecological tumor. Fibroids are highly heterogeneous and can vary in size, quantity, location, stiffness, and symptom severity. Depending on fibroid developmental stage, fibroids can range in cellularity, proliferation, ECM content, and vasculature.^33^ Interestingly, fibroids can increase by up to 135% in volume in 6 months^34^ but have a low mitotic index.^35^ Approximately 11 million individuals are currently diagnosed with fibroids, with an additional 3.7 million individuals with suggestive symptoms who are undiagnosed.^36^ An estimated 25–50% of these people with fibroids experience symptoms, where African American individuals who develop fibroids do so at an earlier age and have more severe symptoms.^37^

Despite fibroid prevalence, there is no cure for fibroids. Treatment options for fibroids are based on symptom severity, fibroid size and location, and the patient’s desire to preserve fertility. The most common intervention includes myomectomy and uterine artery embolization as they are fertility-sparing surgical options. Both options have risks of fibroid recurrence after treatment.^38,39^ Commonly prescribed pharmacologic treatments, including GnRH analogs and SPRMs, are accompanied by a host of side-effects and safety concerns for long-term use.^40,41^ Depending on severity of symptoms, patients also opt to undergo hysterectomy, which remains the definitive treatment for fibroids.^42^ In fact, fibroids are the leading cause of hysterectomy, accounting for 39% of all hysterectomies, annually.^43^ Despite ongoing research efforts, the growth mechanism of fibroid development is not well characterized.^44^ It is imperative that fibroid research articulates why fibroids occur and how to mitigate their growth to better treat and prevent fibroid formation.

It is challenging to study uterine fibroid growth and development as most interventions with fibroids only occur at severe onset of symptoms once fibroids are already well established. Additionally, procurement of samples only occurs following surgical intervention, limiting early stage access and assessment of fibroids.

Since studying fibroid development in humans is challenging, scientists have leveraged in vivo models to study spontaneous fibroid development. The Eker rat remains the best characterized preclinical model for investigating fibroid development and progression as they give rise to fibroid-like tumors.^45–47^ However, inherent differences between rat and human reproductive physiology limit the direct translation of findings. Rats do not naturally menstruate which may pose limitations in modeling fibroids given their link to sex hormones and reproductive age.^48–50^ With these limitations of the Eker rat, there is a critical need for models that more closely mimic human uterine fibroid development. Advantageously, in vitro models can attempt to mimic the complex pathophysiology of uterine fibroids.

As an alternative to in vivo modeling, researchers study isolated fibroid cells from patient samples and fibroid cell lines in 2D. In general, 2D studies involve cell culture on tissue culture plastic, which has a stiffness greater than 2 GPa.^51,52^ Myometrial tissue stiffness has been reported in literature to range between 0.5 and 5 kPa.^16,53^ Fibroid tissue has been reported between 4 and 20 kPa, and in some studies up to 40 kPa.^16,54^ Schutte et al. employed 2D collagen-coated polyacrylamide substrates with stiffnesses varying from 0.2 to 64 kPa and observed slower growth rates in fibroid cells compared to myometrial cells. The fibroid cells spread out with larger 2D surface area compared to myometrial cells on all stiffnesses, and average cell surface area of both cell types tended to increase with stiffness.^55^ This finding was consistent with findings from Norian et al., who observed increasing cell surface area as substrate stiffness increased from 7 to 140 kPa. At the 7 and 30 kPa stiffnesses, fibroid cells appeared more spread with a larger surface area compared to myometrial cells. However, at 75 and 140 kPa, they saw the opposite effect, where the fibroid cells appeared more elongated with a larger surface area.^56^ Taken together, cell mechanosensing may be altered or attenuated in fibroids.

Given the limitations of 2D studies, researchers have turned toward implementing 3D cell culturing techniques to better understand the complexity of fibroids. Recent attempts at 3D fibroid studies largely rely on spheroid formation of fibroid and/or myometrial cells. In 2012, Malik and Catherino suspended fibroid and myometrial cells in 2 mg/mL rat tail collagen (roughly 9 Pa^57^), and the cells maintained spindle morphology and increased collagen I and fibronectin gene expression upon 10 ng/mL TGF-β3 treatment for 3 days.^24^ Banerjee et al. suspended myometrial and fibroid stem cells in Matrigel. They observed spheroids that responded to estrogen and progesterone treatment via size differentials, proliferation markers, and profibrotic markers.^25^ Pellegrino et al. confirmed the formation of fibroid and myometrial spheroids in agarose and in bioprinted alginate matrix.^26^ Commonly used materials, like Matrigel and collagen, have the same limitations of mechanical stiffness, as they are much softer than fibroid tissue. Additionally, there is limited control over spheroid reproducibility, cell migration, and nutrient diffusion.

Here, we established a PEG-based hydrogel to more closely mimic healthy myometrial and fibroid tissue bulk mechanics. Using the PEG-based hydrogel, we can incorporate cells and ECM components that exist in fibroids. Due to their role in generating ECM within fibroids, we used human uterine fibroblasts (HUFs) to model their activation and ECM production. Fibroblasts comprise a significant proportion of the cellular composition of fibroids. Vimentin positive fibroblasts comprise approximately 20–65% of the cellular composition of fibroids.^58^ Given their presence in the fibroid microenvironment, we initially considered fibroblasts in the current scope of this study. To allow for visualization of individual cell bodies and remain consistent with literature seeding densities,^59–62^ we chose to use 4 × 10^6^ cells/mL as our fibroblast seeding cell density.

Varying the concentration of PEG–PQ–PEG (collagen I-derived) and PEG–RGDS (fibronectin-derived) macromers in the hydrogel matrix allows for the modulation of mechanical stiffness and cell adhesion.^32^ Given the patient-to-patient variability in healthy myometrial and fibroid tissue stiffnesses, it is challenging to capture the full physiological stiffness ranges. However, modeling defined soft and stiff conditions enables controlled comparisons that reflect physiologically relevant differences. The observed decreases in matrix stiffness over time are likely due to enzymatic degradation of the PQ peptides, which allows for cell spreading, migration, and matrix remodeling and deposition. Ultimately, despite these decreases, the 4% PEG–PQ–PEG hydrogels and 6% PEG–PQ–PEG hydrogels can be used to mimic physiologically relevant ranges of myometrial and fibroid tissues, respectively.

TGF-β activates fibroblasts to express a αSMA and secrete ECM proteins. Of the TGF-β isoforms, TGF-β3 is suspected to play the most critical role in uterine fibroid pathophysiology since it is elevated in fibroids compared to healthy myometrium.^10,17,63,64^ Serum level elevation of TGF-β3 has been identified as a risk factor for fibroid occurrence.^65^ Given the increased production of ECM proteins in the fibroid microenvironment, researchers believe that TGF-β3 is a mediator of fibroid growth, but its exact role has yet to be elucidated in terms of serving as an initiating factor or as a downstream effect. In 2D studies, it was found that TGF-β3 elicited a fibrotic phenotype of both fibroid and myometrial cells, marked by increases in proliferation and collagen and fibronectin expression.^63,66^ However, TGF-β3 treatment of myometrial cells has also demonstrated antiproliferative effects,^17^ indicating that the effects of TGF-β3 on myometrial cells is perhaps context dependent.^10^

Two-dimensional fibroid studies frequently report TGF-β3 treatment concentrations of 0, 0.1, and 1.0 ng/mL,^12,17,67^ which is representative of TGF-β3 levels in serum.^68^ However, since we aimed to develop a microtissue model of uterine fibroids, we chose TGF-β3 treatment concentrations to reflect that of the bulk fibroid tissue. The concentration of TGF-β3 in fibroid tissue has been reported to be an average of 171.29 ± 91.81 pg/mg, using roughly 100 mg of homogenized tissue samples in 1 mL of PBS.^68^ We estimated the average concentration of TGF-β3 in fibroid tissue to be 17.129 ± 9.181 ng/mL. Therefore, we chose the following concentrations to be representative of TGF-β3 levels in fibroid tissues: 10 ng/mL and 20 ng/mL.

Given that fibroid ECM is primarily composed of collagen—particularly collagen I, followed by collagen III, both of which are significantly elevated compared to myometrial tissue^6,14,69^ —we quantified collagen I (Col1) expression. Elevated Col1 expression with 20 ng/mL TGF-β3 in both myometrial-mimicking and fibroid-mimicking hydrogels align with previous studies of fibroid and myometrial cell production of Col1 upon TGF-β3 treatment in 2D culture and 3D culture in collagen gels.^24,63^ The increase in cell elongation in 3D, consistent with the morphology of myometrial and fibroid cells cultured in 3D collagen gels,^24^ along with increased αSMA and Col1 suggests an activated fibroblast phenotype that mimics the cell morphology and ECM content of fibroids.

We know from literature that fibroid progression involves distinct changes in cellular activity over time. Flake et al. have characterized the fibroid phases based on collagen content and proliferation. They showed that as fibroids progress in phase, as collagen content increases, cell proliferation progressively decreases based on proliferating cell nuclear antigen (PCNA) immunohistochemical staining of fibroid tissues. Observing the maximum staining areas of the fibroid samples, the percentage of PCNA positive cells in early fibroid development was 59.1% compared to 15.5% in a more established phase.^33^ Additionally, Dixon et al. investigated fibroid tissue cell proliferation using PCNA compared to Ki67 staining of 45 fibroids of various sizes isolated from 6 patients. They observed low proliferating cells with both PCNA and Ki67. Interestingly, proliferation was independent of fibroid sizes and exceeded that of all matched myometrium.^70^

Given that proliferation and collagen deposition can benchmark fibroid growth, we quantified HUF cell proliferation over time in our PEG-based hydrogels. In the myometrial-mimicking hydrogels, we observed decreased cell proliferation between 24 h and 4 days with and without TGF-β3 treatment. From 4 to 6 days, we observed an increase in proliferation in the nontreated hydrogels. These transient decreases in proliferation at day 4 may suggest an initial response to matrix-derived cues, which later is reversed by autocrine signaling as the cells secrete additional growth factors that may promote renewed proliferative activity observed at 6 days.^71^ Similarly, in the fibroid-mimicking hydrogels, we saw decreased proliferation in both nontreated and TGF-β3-treated groups from 24 h to 4 days and from 24 h to 6 days. However, on day 6, TGF-β3-treatment significantly reduced proliferation compared to no treatment. Taken together, our data is consistent with literature reports that indicate a reduction in cell proliferation in the fibroid microenvironment with increasing time,^33,70^ mimicking an early fibroid development phase.

To determine whether decreased cell proliferation in our model may indicate a phenotypic switch in fibroblasts from a proliferative state to an ECM-producing state,^72,73^ we also quantified collagen production over time with TGF-β3 treatment using the same time points: 24 h, 4 days, and 6 days. Because collagen I and III show the most pronounced increases in fibroid tissue compared to other collagens, we performed immunocytochemical staining for Col1 and Col3 over time. In the fibroid-mimicking hydrogels, Col1 volume significantly increased with TGF-β3 treatment at each time point. Additionally, there was greater expression of Col1 compared to Col3, which is consistent with in vivo literature findings.^14^ Considering the trends in decreased cell proliferation along with increased collagen with TGF-β3 treatment, these results mimic the phenotypic switch from proliferation to collagen production, recapitulating in vivo fibroid characteristics.

In literature, uterine fibroids have exhibited a metabolic shift, where an increase in glycolysis is observed, enabling rapid cell growth and proliferation.^74,75^ When treated with TGF-β3, the conditioned media from the fibroid-mimicking hydrogels had significantly less TGF-β3 than that from the myometrial-mimicking hydrogels, suggesting greater uptake of the treatment by cells encapsulated in the stiffer matrix. l-Lactate is a major byproduct of aerobic glycolysis and was slightly elevated in conditioned media from the TGF-β3-treated hydrogels. However, with or without TGF-β3, HUFs produced high levels of l-lactate, indicating high glycolytic activity. Together, these findings suggest metabolic activation as seen in vivo, allowing for cell growth and proliferation in the 3D in vitro environment.

In uterine fibroids, high LOX expression correlates with increased collagen cross-linking, ECM stiffness, and αSMA, a marker of fibroblast activation; LOX expression is significantly higher in fibroid tissue compared to myometrial tissue.^76–78^ In our hydrogels, we observed higher LOX expression when treated with TGF-β3. These findings further underscore a metabolically active, fibrotic phenotype of uterine fibroblasts in the myometrial- and fibroid-mimicking hydrogels with enhanced ECM remodeling and collagen cross-linking.

Lastly, we determined whether uterine fibroblast activation in response to TGF-β3 treatment was specifically mediated through TGF-β signaling. To do so, we selected a small molecule TGF-β type 1 receptor inhibitor, SB-431542, that has previously been shown to attenuate TGF-β3 mediated signaling in uterine fibroid cells.^79^ Consistent with these findings, we found that TGF-β inhibition reduced collagen I expression in the fibroid-mimicking hydrogels. Therefore, we can attribute the observed experimental results to TGF-β3 mediation. Ultimately, these findings establish our hydrogel platform as a relevant system to evaluate small molecules that may modulate TGF-β3 signaling for potential fibroid therapeutics.

## 5 Conclusions

Here, we established an in vitro model to characterize uterine fibroblast activation in fibroid- and myometrial-mimicking microenvironments using PEG-based hydrogels. We incorporated collagen- and fibronectin-derived peptides and altered bulk mechanical stiffness to model the ECM components of fibroids and myometrial tissue. We observed that human uterine fibroblasts encapsulated in the PEG-based hydrogel mimicking fibroid stiffness produced higher levels of fibrotic activation markers in response to TGF-β3, including elevated αSMA, collagen I, and cell elongation. We also quantified fibroblast proliferation and collagen I and III production over time; while proliferation decreased in the hydrogels mimicking fibroid stiffness, and collagen I was upregulated in fibroblasts when treated with TGF-β3 at 24 h, 4 days, and 6 days. In both the myometrial- and the fibroid-mimicking matrices, fibroblasts demonstrated high glycolytic activity, and TGF-β3 stimulation upregulated LOX expression, further indicating fibrotic activation. Lastly, collagen I expression was reduced upon inhibition of TGF-β signaling, demonstrating that the observed effects are driven through the TGF-β pathway and underscoring that this platform could be used to screen potential therapeutics.

There are several limitations of this work. The cellular composition of fibroids is highly variable with common cell types including smooth muscle cells, fibroblasts, endothelial cells, and immune cells.^80^ Building upon this work, we can systematically explore additional aspects of the fibroid environment, including the contribution of uterine smooth muscle cells and immune cells,^77^ as well as other critical and emerging modulators of fibroid growth, such as inflammatory factors and vitamin D.^44,81^ A logical next step would be to coculture fibroblasts with smooth muscle cells and replicate key fibroid cell types, given the smooth muscle cell prevalence in the fibroid microenvironment. In addition, this work used primary uterine fibroblasts isolated from one donor, which may not capture the biological variability that may exist across patients; as such, results from this study may not fully represent all fibroblast behaviors observed in the broader population. Lastly, we characterized uterine fibroblast ECM production and remodeling, proliferation, and metabolic activity in response to TGF-β3 in the hydrogels. Further characterizations of fibroblast activation could include assessing fibroblast contractility via comprehensive analysis of actin and myosin filaments.

Ultimately, this work aimed to develop a 3D in vitro model to investigate how uterine fibroblasts respond to fibrotic cues such as TGF-β3 in hydrogels that recapitulate myometrial and fibroid tissues, allowing further investigation into the cellular mechanisms governing fibroid development and progression. Through these studies, we have demonstrated the ability to evaluate human uterine fibroblasts in physiologically relevant PEG-based hydrogels that mimic fibroids. Future studies aim to use this model to elucidate fibroid pathogenesis and screen potential therapeutics in order to target fibroid growth.

## Supplementary Material



## Notes

Representative images of immunostaining controls without primary antibodies (PDF)
