# Supplementary material for: Modeling Uterine Fibroids Using Bioengineered Hydrogels
Source: ACS Biomater Sci Eng. 2025 Sep 11;11(10):6068–83. doi: 10.1021/acsbiomaterials.5c01026 (PMC12525010; doi:10.1021/acsbiomaterials.5c01026)
Supplement: Supplementary file 1 [file ab5c01026_si_001.pdf]

Modeling Uterine Fibroids Using Bioengineered Hydrogels

Allison K. Moses, Miriam Tamaño-Blanco*, and Erika Moore**

*Fischell Department of Bioengineering, University of Maryland, College Park, Maryland, United States.

Corresponding Author

Erika Moore – Fischell Department of Bioengineering, University of Maryland, College Park, Maryland 20742, United States; orcid.org/0000-0003-2192-6147; Email: emt@umd.edu

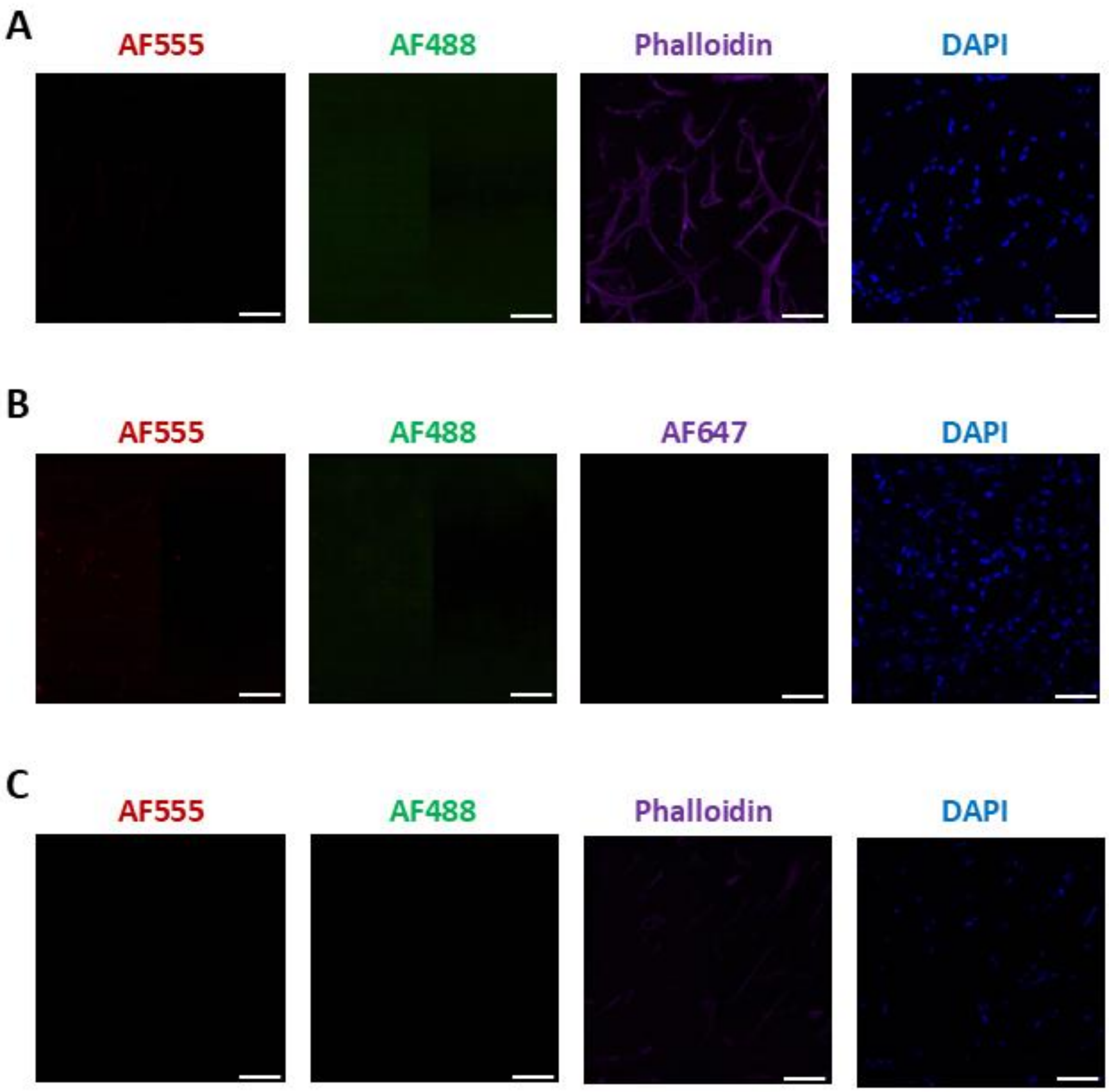

Supplementary Figure 1: Representative images of immunocytochemistry staining controls without primary antibodies. Primary antibodies had little to no nonspecific staining. (**A**) Immunostaining panel for αSMA, Col1, and cell bodies with or without TGF-β treatment of HUFs, where the following fluorescent channels are imaged: αSMA (AF555), Col1 (AF488), Phalloidin (AF647), and DAPI. (**B**) Immunostaining panel for HUF production of Col1, Col3, and Ki67 over

time with or without TGF-β treatment of HUFs, where the following fluorescent channels are imaged: ColIII (AF555), Col1 (AF488), Ki67 (AF647), and DAPI. (**C**) Immunostaining panel for HUF production of αSMA, Col1, and cell bodies with TGF-β3 treatment and/or TGF-β inhibition, where the following fluorescent channels are imaged: αSMA (AF555), Col1 (AF488), Phalloidin (AF647), and DAPI. All scale bars = 100 µm; 20x magnification.
